# Occlusal force predicts global motion coherence threshold in adolescent boys

**DOI:** 10.1186/s12887-018-1309-2

**Published:** 2018-10-18

**Authors:** Kensuke Kiriishi, Hirokazu Doi, Nobuaki Magata, Tetsuro Torisu, Mihoko Tanaka, Makoto Ohkubo, Mitsuhiro Haneda, Masaki Okatomi, Kazuyuki Shinohara, Takao Ayuse

**Affiliations:** 1 Department of Clinical Physiology, Graduate School of Biomedical Sciences, Nagasaki University, Nagasaki, Japan; 2 Graduate School of Biomedical Sciences, Nagasaki University, Nagasaki, Japan; 3 Football club BRISTOL, Nagasaki, Japan; 4 Department of Prosthetic Dentistry, Graduate School of Biomedical Sciences, Nagasaki University, 1-7-1 Sakamoto, Nagasaki, Japan

**Keywords:** Masticatory stimulation, Occlusal force, Bite force, Global motion coherence, Aerobic capacity, Adolescence

## Abstract

**Background:**

Beneficial effects of mastication on cognitive abilities in the elderly have been shown in human studies. However, little is currently known about the effect of masticatory stimulation on cognitive and perceptual ability in younger populations. The purpose of the present study is to investigate the influences of masticatory stimulation on perceptual ability in adolescent boys.

**Methods:**

The present study examined the relationship between occlusal force (i.e., masticatory stimulation) and visual perception ability in adolescent boys. Visual perception ability was quantified by measuring global motion coherence threshold using psychophysical method. As an index of masticatory stimulation, occlusal force was measured by pressure sensitive film. We also measured participants’ athletic ability, e.g. aerobic capacity and grip strength, as potential confounding factor.

**Results:**

The multiple regression analysis revealed a significant negative correlation between global motion coherence threshold and occlusal force, which persisted after controlling for confounding factors such as age and aerobic capacity.

**Conclusions:**

This finding indicates that masticatory stimulation enhances visual perception in adolescent boys, indicating the possibility that beneficial effects of masticatory stimulation are observed not only in the elderly but in developing population consistently with the findings of the previous animal studies.

## Background

Masticatory stimulation, i.e.*,* occlusal sensation induced by chewing movements, transmits various information to the central nervous system (CNS) [1]. After reaching the periodontal membrane, masticatory stimulation is transmitted to the CNS via the mesencephalic trigeminal nucleus [2]. The CNS then uses this information for object identification through texture and hardness [1, 3].

Recent studies have revealed functional linkage between mastication and cognitive function during the growth period [4–6]. These studies have also pointed out that neuronal development might be affected by masticatory stimulation in the growth period as defined by Scammon’s curve. For example, rodents fed only soft food during the growth phase have been reported to show impaired performance in behavioral tasks such as conditioned avoidance learning and the maze learning test, compared with rats fed solid food [7, 8]. Because these tasks require functionality of the hippocampus and prefrontal area, the results suggest that increased masticatory stimulation induced by chewing solid foods facilitates the development of the mnemonic and navigational functions of these neural regions. Likewise, feeding only on soft foods has been shown to decrease hippocampal formation and to precipitate hippocampal neural cell apoptosis in mice [9–12].

In humans, neuroimaging studies have shown increased activation in several neural regions, including the primary/supplementary motor area, cerebellum, somatosensory areas, thalamus, insula, and hippocampus, during chewing movement compared with control movement [13–15]. These and results from other studies support the view that mastication also enhances CNS functions in humans [16–22]. However, previous human studies have only investigated associations between masticatory stimulation and a limited variety of cognitive functions such as long-term memory [19] and arousal regulation [15, 22, 23]. Thus, whether masticatory stimulation also facilitates other important function such as visual perception remains unclear. In addition, the majority of existing studies have focused on the effects of mastication in the elderly [18, 20, 21], with few having paid attention to developing populations, despite accumulating evidence from animal studies that masticatory stimulation influences performance in learning and emotional response during the growth period as early as the early postnatal stage [5].

The goal of the present study is to investigate the effects of masticatory stimulation on visual perception ability in a human developing population. To achieve this, we examined the association between visual perception ability and occlusal force in adolescent boys. We recruited adolescent boys belonging to an amateur football club. The reasons for recruiting this particular group were two-fold. First, acquisition of a sophisticated level of cognitive and visual perception ability is adaptive for football players. Previous studies have reported that football players require flexibility in control of visual attention and decision-making based on incoming sensory information during a football match [24, 25] in order to respond to a dynamically changing, unpredictable and externally paced environment [26, 27]. Second, Ohkawa et al. [28] revealed strong masticatory muscle activities during kicking in football players. Neuroimaging studies have shown that brain structure is still immature and highly plastic to external inputs during adolescence [29, 30]. We thus surmised that prominent influences of mastication/occlusion on perceptual ability should be observed in boys who routinely play football during this developmental stage due to abundant input of masticatory stimulation into the CNS.

Visual perception ability has been quantified by measuring global motion coherence [31–33]. The reason for measuring this particular ability of visual perception is that perception of global motion information is of great importance when playing football. In judging game situations, players have to rely on global patterns of the direction of movement of other players. We thus thought that we could quantify one aspect of visual perception ability that is adaptively important for football players by measuring global motion coherence threshold.

On the basis of previous studies linking masticatory stimulation and enhanced CNS activation [13, 16–21] together with protracted development of global motion perception ability into late childhood [32, 34], we hypothesized that adolescents with high occlusal force, whose CNS would supposedly receive abundant masticatory stimulation, should show superior global motion coherence perception to adolescents with low occlusal force.

Children with greater aerobic capacity have been shown to exhibit superior performance in cognitive functions such as learning, recognition, and memory compared to those with lower aerobic capacity [35–37]. We therefore measured aerobic capacity as a potential confounding factor. A spurious association between visual perception ability and occlusal force might emerge because both of them develop with age. Furthermore, even if there is actually relationship between these variables, it remains unclear whether visual perception ability is specifically linked to occlusal force, hence masticatory stimulation; it remains possible that visual perception ability is associated with general physical ability and not specifically linked to occulusal force. To exclude these possibilities, we also included age and the measures of anaerobic aspect of athletic ability, i.e. shuttle run record and grip strength, as confounding factors.

## Methods

### Participants

In total, 51 early- to middle-adolescent boys aged 9–15 years old (Mean (M) = 12.1 years; standard deviation (SD) = 1.7 years) were recruited from an amateur football club in Japan. All had normal or corrected-to-normal visual acuity and none reported problems in observing the visual stimuli used in the present study. Children with the history of psychiatric condition or currently taking mediation were excluded form the present study. All procedures performed in this study were in accordance with the ethical standards of the institutional research committee and with the 1964 Helsinki declaration and its later amendments or comparable ethical standards.The guardians and children were provided with information about the research and all gave written informed consent prior to enrolment.

### Procedure

The following measurements were taken from each participant: global motion coherence threshold, occlusal force, and athletic ability. Dental development was also evaluated by observing current situation of number of adult teeth and deciduous teeth.

#### Global motion coherence threshold measurement

**Fig. 1:**
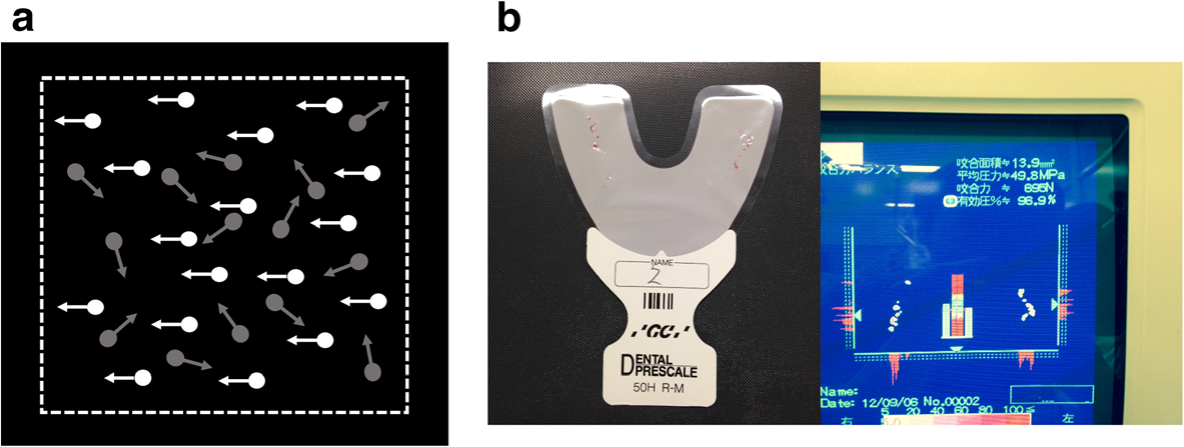
**a** Schematic representation of stimulus in global motion coherence threshold measurement. Coherently moving dots are presented in white, while the remaining dots are in gray. Arrows indicate the direction of dot movement. Arrows and dotted line depicting the boundary of dot movement were not shown in the actual experiment. **b** A sample of colored film (left) and the measurement system (right) used in occlusal force measurement

Global motion coherence threshold was measured using a laptop computer with a 17-in. display viewed from roughly 40-cm away. During each trial, after presentation of the fixation cross for 250 ms, 100 white dots moving at a speed of 60 pixel/s appeared within an invisible square about 16.8 cm in width at the center of the screen for 400 ms, with *p*% of the 100 dots moving in the same direction, either to the right or left horizontally, while the remaining dots moved in random directions. The lifetime of each dot was 200 ms, after which they were relocated to a random location within the invisible square. Relocation of dots occurred asynchronously. The lifetime was imposed to prevent participants from adopting the strategy of continuously tracking only a handful of dots rather than focusing on the global pattern. The concept of stimulus display is schematically shown in Fig. 1a. Participants indicated the direction of motion by key-press after the disappearance of white dots. When a participant did not respond within 10 s of the disappearance of a stimulus, an incorrect response was considered to have been made. The proportion *p* of coherently moving dots in each trial was determined using a staircase procedure [38]. The staircase started from *p* = 100 to help participants grasp the concept of the task. This proportion decreased when the participant made correct responses three times consecutively, and increased when the participant made an incorrect answer. Step size started from 32% and halved at every reversal. The threshold was analyzed on the basis of the last 6 reversals.

#### Occlusal force measurement

Occlusal force (N) and occlusal contact area were evaluated using the Dental Prescale system (Fuji Film, Tokyo, Japan), in which a pressure-sensitive film (50H type) shows color variation depending upon the force of the applied occlusal pressure within a range of 5–120 MPa. We measured the occlusal force of all participants using a medium-sized dental prescale. Each subject was requested to bite a prescale film in the centric occlusal position for 2 s at maximum force with deeply sitting position. During measurement, his head was aimed at a position in which Frankfurt plane was parallel to the floor. Participants were taught by a dentist (K.K.) how to bite in a centric occlusal position so that jaw position did not affect occlusal state. Films were scanned using an Occluzer FPD-703 scanner™ (GC International, Tokyo, Japan) [39]. Examples of films with coloration due to applied occlusal pressure, and the scanner used are shown in Fig. 1b. The discolored area was recorded as the occlusal contact area. Maximum occlusal force was determined based on the degree of coloration and the area of each contact point.

#### Occlusal support

A licensed dentist (K. K.) evaluated tooth occlusal support for each participant based on Eichner’s classification as a reference. Eichner’s classification is used as an evaluation of the occlusion support level widely and popular in occlusal force studies [40–42]. Because it might be inappropriate to adapt Eichner’s classification to assessment of children’s mixed dentition, we decided to evaluate occlusal support areas using modified classification in which an occlusal support area is defined as a minimum of one upper and one lower tooth in maxillomandibular contact in the premolar or the molar region on the left or right sides. According to the number of occlusion support areas, maxillomandibular occlusal states were divided into the following three groups in this study (four supporting areas, three supporting areas, two supporting areas).

#### Evaluation of tooth development

Dental ages of participants were determined based on Hellman’s dental development stages by a licensed dentist (K.K.). Division of Hellman’s dental developmental stages is based on dental age and clinical finding [43]. Dental developmental stages were divided into groups as follows: IA, deciduous tooth non-eruption phase; IC, deciduous tooth eruption starting phase; IIA, completion of primary occlusion; IIC, eruptive phase of permanent first molar or incisor; IIIA, eruption of permanent first molars or incisors completed; IIIB, exchange phase of lateral teeth; IIIC, eruptive phase of permanent second molar; and IVA, eruption of permanent second molar completed.

#### Athletic ability measurement

##### Grip strength

To measure grip strength, we used a Smedley-type hand dynamometer (T.K.K.5001, Takei Scientific Instruments Co.,Ltd., Nagoya, Japan). We instructed the participant to grip the hand dynamometer as firmly as possible with legs spread and arms lowered naturally. We prohibited the participant from swinging the hand dynamometer around. Grip strengths of both left and right hands were each measured twice, then averaged.

##### Aerobic capacity

The aerobic capacity of each participant was measured using a maximal multistage 20-m shuttle-run test [44–46]. This required the participant to run between parallel lines drawn on the ground 20-m apart. The timing for participants to reach each line and turn was set by a beep sound played by a CD player. The interval between each beep was shortened so that running speed increased by 0.5 km/h every minute. The test continued until the participant was unable to maintain the designated running speed. We estimated maximum oxygen consumption (VO_2_max) based on the total number of turns made by referring to the “20-m shuttle run (round trip endurance running) maximum oxygen consumption estimation table” issued by the Ministry of Education, Culture, Sports, Science and Technology of Japan.

##### 50-m run

We measured the individual record time for the participant to run 50 m on the football ground for the 50-m run for each participant. The record was measured to the nearest 1/10th of a second, then rounded up to the next whole number.

### Statistical analysis

First, we tested whether the distribution of each variable conforms to normal distribution. Shapiro-Wilk normality test revealed signfiicat deviation from normal distribution in age, grip strength and 50 m run record (Ws < .94, ps < .05). These variables were submitted to further analyses after being transformed by Box-Cox transformation [47].

In the second step of analysis, we examined bivariate correlations between every pair of measurements in addition to computing descriptive statistics. Due to the explanatory nature of this analysis, we did not control for the family-wise error rate. The third set of analyses specifically focused on examination of our primary hypothesis, that high masticatory stimulation would facilitate visual perception ability. To achieve this, we carried out multiple regression analysis with global motion coherence threshold as the dependent variable. Independent variables included occlusal force, age, grip strength, aerobic capacity and 50-m run record. We detected no multicollinearity by the criteria of variance inflation factor (VIF) > 10. Thus, all the independent variables were entered into the model. All statistical analyses were conducted using R software (The R Foundation).

## Results

Data from 3 participants were discarded from the final analyses owing to a failure to obtain measurements on occlusal force and athletic ability (*n* = 2), or broken teeth (*n* = 1). Data from a further 10 participants were excluded from the final dataset because the data for global motion coherence threshold measurement was missing due to equipment failure (*n* = 6), or the individual showed poor performance due to inattention or misunderstanding of instructions (*n* = 4). The final dataset included the data from 38 participants between 9 and 15 years old (M = 12.2 years; SD = 1.8 years). The final sample size is comparable to existing studies on the correlation between visual perception and motor skills in pediatric samples [48, 49]. Participants included in the final dataset were divided into three classes according to occlusal support:four supporting areas (*n* = 24), three supporting areas, (*n* = 5), and two supporting areas (*n* = 9). Participants in the final dataset comprised three groups according to Hellman’s dental development stage: group IIIB (*n* = 16), group IIIC (*n* = 19), and group IVA (*n* = 3), indicating that the majority of participants were still in the tooth eruption phase. Mean and standard deviation of measured variables are summarized in Table 1.

**Table 1:** Means and standard deviations of measurements

	Mean	SD
Contact Area (mm^2^)	9.18	3.64
Mean Pressure (MPa)	63.39	78.46
Occlusal Force (N)	463.82	177.62
Grip Strength (kg)	21.99	7.78
Aerobic Capacity (ml/kg・min)	48.29	4.91
50 m Run Record (sec)	8.07	0.91
GMC Threshold (%)	54.48	22.11

Note. *SD* Standard Deviation, *GMC Threshold* Global Motion Coherence Threshold

### Exploratory bivariate correlation analysis

**Fig. 2:**
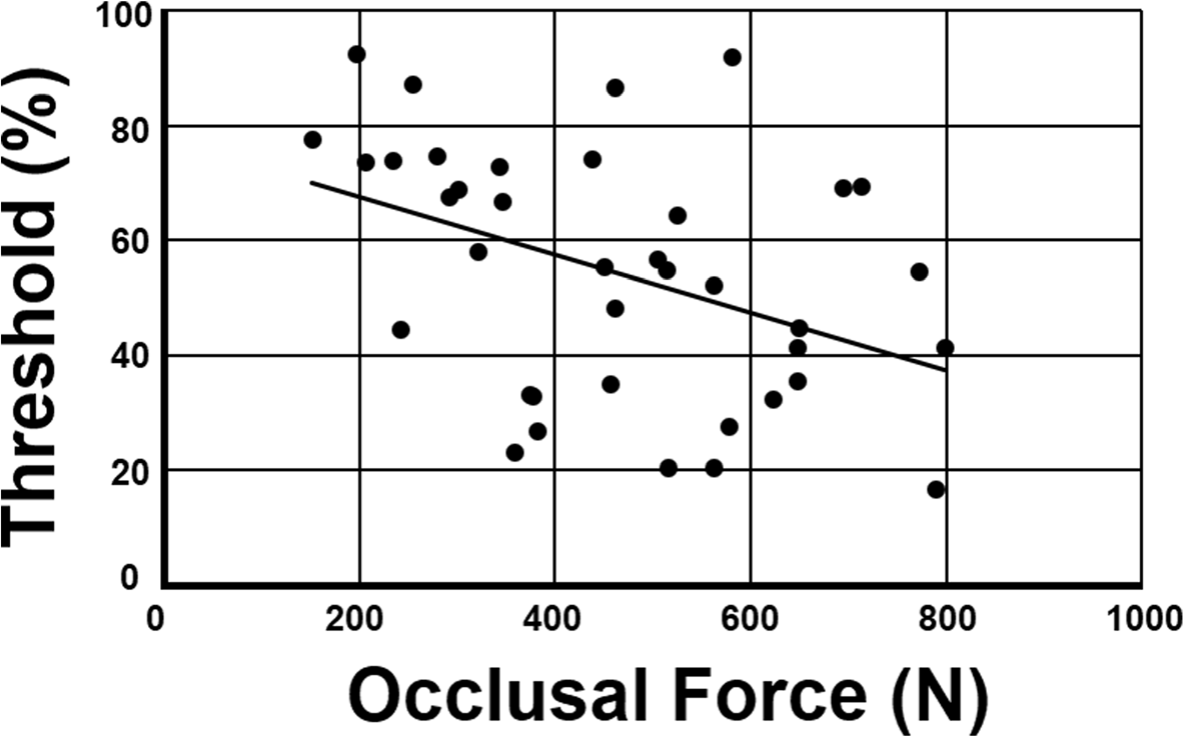
Scatterplot showing relationship between occlusal force and global motion coherence threshold

We first investigated the relationship among variables in an exploratory manner by testing bivariate correlations between every pair of variables. Results are summarized in Table 2. As predicted, a significant correlation existed between occlusal force and global motion coherence threshold (*r* (36) = − 0.415, *p* = .0096). The scatter plot for occlusal force and global motion coherence threshold is shown in Fig. 2. Aside from this, 50-m run record correlated negatively with age (*r* (36) = − 0.811, *p* < .001), grip strength (*r* (36) = − 0.859, *p* < .001) and aerobic capacity (*r* (36) = − 0.651, *p* < .001). Aerobic capacity showed significant positive correlations with age (*r* (36) = 0.756, *p* < .001) and grip strength (*r* (36) = 0.552, *p* = .0003). A significant positive correlation was also seen between grip strength and age (*r* (36) = 0.835, *p* < .001).

**Table 2:** Correlation coefficients between each pair of observed variables

	Occlusal Force	Age	Grip Strength	Aerobic Capacity	50 m Run Record	GMC Threshold
Contact Area	0.961**	0.014	0.135	0.023	−0.034	− 0.331*
Occlusal Force		0.028	0.165	0.038	−0.089	−0.415**
Age			0.835**	0.756**	−0.811**	−0.294
Grip Strength				0.552**	−0.859**	−0.251
Aerobic Capacity					−0.651**	−0.228
50 m Run Record						0.159

Note.*p < .05, **p < .01 without family-wise error rate control*. GMC Threshold* Global Motion Coherence Threshold

### Multiple regression analysis

To examine the nature of the significant correlation between occlusal force and global motion coherence threshold in greater detail, we conducted a multiple regression analysis with global motion coherence threshold as the dependent variable. Predictors included occlusal force, age, averaged grip strength, aerobic capacity, and 50-m run record. The standardized coefficient (*β*) of each predictor and associated statistics are summarized in Table 3. Occlusal force, but not aerobic capacity, correlated significantly with global motion coherence threshold after controlling for confounding factors (*β* = − 0.424, *t* = − 2.72, *p* = 0.0104, *df* = 32). The same analysis was run on a subset of data excluding participants with only three or two supporting areas. That analysis revealed a significant influence of occlusal force on global motion coherence threshold (*β* = − 0.599, *t* = − 3.43, *p* = 0.003, *df* = 18) after controlling for the effects of confounding variables.

**Table 3:** Standardized coefficients and statistics in multiple regression analysis

	β	SE	*t*-value	*p*-value
Intercept	<.0001	0.147	<.0001	>.999
Occlusal Force	−0.424	0.156	−2.724	0.010*
Grip Strength	−0.029	0.361	−0.08	0.937
Aerobic Capacity	−0.031	0.245	−0.129	0.899
50 m Run Record	−0.332	0.323	−1.027	0.312
Age	−0.503	0.364	−1.383	0.176

Note. *Grip Strength* Averaged Grip Strength, *Shuttle Run* Shuttle-Run Record*. df* = 32, *p < .05*. SE* Standard error

## Discussion

The present study investigated associations between occlusal force as an indicator of masticatory stimulation, and visual perception ability in adolescent boys playing football. The main finding was that global motion coherence threshold correlated negatively with occlusal force, indicating that children with stronger occlusal force exhibited higher sensitivity to global motion coherence, in line with our hypothesis. The association even persisted after controlling for confounding factors such as age and aerobic capacity by multiple regression analysis. The association between occlusal force (i.e., masticatory stimulation) and global motion coherence persisted when considering only data from participants with four supporting areas, indicating that the observed relationship was not an artifact due to missing support zones. Chewing and masticatory stimulation have been suggested to enhance mnemonic function in middle-aged adults and the elderly [15, 18, 20, 21]. To the best of our knowledge, this represents the first study to indicate a facilitatory effect of masticatory stimulation on perceptual ability in developing humans.

Although exactly how masticatory stimulation (occlusal force) may enhance cognitive function in humans during the developmental period has not been clarified, Fukushima-Nakayama et al. [4] indicated in an in vivo study that neuronal functions underlying memory and learning might be affected by changes in masticatory stimulation during the growth period. They further revealed that changes in masticatory stimulation can modulate neurogenesis and neuronal activity in the hippocampus, functionally contributing to cognitive function in the growth period. Likewise, Nose-Ishibashi et al. [5] showed that poor masticatory stimulation induced by soft-food feeding during the post-weaning period led to weaker pre-pulse inhibition in mice, i.e., a behavioral indicator of increased vulnerability to psychiatric conditions. Interestingly, this behavioral impairment was accompanied by reduced cell proliferation in hippocampal regions. Given these findings together with the finding from a human neuroimaging study that visual perception of global motion stimuli recruits the hippocampal region [50, 51], one potential interpretation of the present results is that enhanced hippocampal function induced by masticatory stimulation led to higher ability of global motion coherence perception in adolescent boys.

At the same time, facilitatory effects of masticatory stimulation on neural regions other than hippocampus may be equally plausible for explaining the present finding. Takeda and Miyamoto [14] have shown increased activation in fronto-parietal regions while chewing gum than during chewing movement without masticatory stimulation. Likewise, Sakamoto et al. [52] revealed faster elicitation of event-related potential peaks during auditory processing task after chewing than control mouth movement (see also [22]). These studies indicate the possibility that masticatory stimulation enhances functionality in widespread neural regions. Frota de Almeida et al. [6] revealed that long-term reduction of masticatory stimulation leads to astrocyte hypoplasia in 3-month-old mice. Considering that astrocytes play pivotal roles in regulating cerebral blood flow [53, 54], proliferation of astrocytes facilitated by stronger masticatory stimulation during growth period may induce better control of blood flow distribution in CNS, thereby enhancing cognitive and perceptual abilities.

Occlusal force as an index of masticatory function has been shown to be affected by maxilla-mandibular occlusal state [55, 56]. Because the present study recruited adolescent children, developmental stage could conceivably have influenced both occlusal state and perceptual ability, thereby yielding a spurious association between variables. In the occlusal force measurements of the present study, a dentist instructed each participant to bite in the centric occlusal position so that jaw position did not affect occlusal state. In addition, dental age evaluation confirmed that all teeth grew normally in participants whose data were included in the final analysis. Further, the correlation between occlusal force and global motion coherence threshold was maintained after controlling for the effect of age or when considering only participants with four supporting areas. Based on these results, we are fairly confident that the observed correlation represents a real effect, not an artifact of developmental stage, jaw position, or occlusal state.

Caution should be exercised in interpreting the data due to the following limitations of the present study. First, the sample size in the present study was relatively small, so the present findings should be replicated in a larger sample size. Second, participants in the present study comprised members of an amateur football club team. Football players experience intense contact and strong occlusal force when performing headshots and shoots [28]. Frequent experience of strong occlusal force might thus have accentuated the effects of occlusal force on visual motion perception in this particular group. Examining whether the present findings can be generalized to a wider population including girls would be an important issue for future study. Interestingly, a recent study reported a positive correlation between football technique level and cognitive function [26]. Determining whether the observed link between visual motion perception and occlusal force is mediated by level of football technique would thus also be interesting. The third limitation is that we did not collect data of DMFT (decayed, missing, and filled teeth) index and anthropometric variales, e.g. body weight and body height. Although they were not of the primary interest in the present study, it is still conceivable that these variales moderate the observed association between occlusal force and global motion coherence threshold.

## Conclusion

The present study revealed a negative correlation between occlusal force and global motion coherence threshold, which indicates that masticatory stimulation possibly enhances visual perception ability not only in the elderly, but also in adolescent children. At the same time, it remains equivocal whteher the present finding can be generalized to wider population including girls as well. Thus, further study is required to establish the association between masticatory stimulation and perceptual ability in developing population.

## Abbreviations

CNS: Central nervous system
DMFT: Decayed, missing, filled teeth
GMC: Global motion coherence
M: Mean
SD: Standard deviation
SE: Standard error
